# Metabolomics analysis of grains of wheat infected and noninfected with *Tilletia controversa* Kühn

**DOI:** 10.1038/s41598-021-98283-3

**Published:** 2021-09-23

**Authors:** Zhaoyu Ren, Mingke Fang, Ghulam Muhae-Ud-Din, Haifeng Gao, Yazhen Yang, Taiguo Liu, Wanquan Chen, Li Gao

**Affiliations:** 1 State Key Laboratory for Biology of Plant Disease and Insect Pests, Institute of Plant Protection, Chinese Academy of Agricultural Sciences, Beijing, China; 2 School of Agriculture, Yangtze University, Jingzhou, China; 3 Institute of Plant Protection, Ministry of Agriculture and Rural Affairs, Xinjiang Academy of Agricultural Sciences, Urumqi, China

**Keywords:** Microbiology, Plant sciences

## Abstract

Dwarf bunt caused by the pathogen *Tilletia controversa* Kühn is one of the most serious quarantine diseases of winter wheat. Metabolomics studies provide detailed information about the biochemical changes at the cell and tissue levels of plants. In the present study, a liquid chromatography/mass spectrometry (LC/MS) metabolomics approach was used to investigate the changes in the grain metabolomics of infected and noninfected with *T. controversa* samples. PCA suggested that *T. controversa*-infected and noninfected samples were separated during the interaction. LC/MS analysis showed that 62 different metabolites were recorded in the grains, among which a total of 34 metabolites were upregulated and 28 metabolites were downregulated. Prostaglandins (PGs) and 9-hydroxyoctadecadienoic acids (9-HODEs) are fungal toxin-related substances, and their expression significantly increased in *T. controversa-*infected grains. Additionally, the concentrations of cucurbic acid and octadecatrienoic acid changed significantly after pathogen infection, which play a large role in plant defense. The eight different metabolic pathways activated during *T. controversa* and wheat plant interactions included phenylalanine metabolism, isoquinoline alkaloid biosynthesis, starch and sucrose metabolism, tyrosine metabolism, sphingolipid metabolism, arginine and proline metabolism, alanine, aspartate, and glutamate metabolism, and tryptophan metabolism. In conclusion, we found differences in the metabolic profiles of wheat grains after *T. controversa* infection. To our knowledge, this is the first study to evaluate the metabolites in wheat grains after *T. controversa* infection.

## Introduction

Wheat is one of the most important staple crops and plays a fundamental role in food security worldwide^1^. Wheat production is often negatively affected by infection from a wide variety of pathogens. Wheat dwarf bunt (caused by the fungal pathogen *Tilletia controversa* Kühn) is a destructive disease that causes significant quality and quantity losses in wheat growing regimes^2^. Metabolomics is an omics technology that can comprehensively evaluate small, endogenous molecules (such as nucleotides, organic acids, sugars and amino acids) to analyze the interrelationships between genetic structure, gene expression, protein function, and environmental impact^3^. These compounds are the substrates and byproducts of cell processes (such as enzymatic reactions) and, as such, have a direct effect on phenotypes. The subdiscipline of plant metabolomics is a growing body of research in the plant–microbe interaction area^4,5^. Plant defense mechanisms can be regulated by metabolomics in fungus-infected plants. Plants have evolved different mechanisms to counterattack pathogen infection, including several layers of inducible and constitutive defenses, biochemical molecules and different metabolites^6,7^. Metabolites are the end products of translation and transcription; therefore, changes in metabolite abundance may be regarded as a major feature of plant interactions with pathogens and the environment^3,8^. Previous studies have shown that wheat metabolites were different in *Fusarium graminearum*-infected plants compared with normal plants^9^. *Magnaporthe oryzae* altered the alanine contents in rice compared to normal rice leaves^10^. Doehleman^11^ investigated the metabolites in maize tumors after *Ustilago maydis* infection. Similarly, metabolic profiling strategies were used to determine the mechanisms of plant defense against *Rhizoctonia solani* in soybeans, rice, lettuce, and potatoes^12–15^. Metabolomics analysis has been performed in *Botrytis. cinerea-*infected tomato, strawberry, *Arabidopsis* and grape plants^16,17^. Gas chromatography-mass spectrometer (GC–MS) based metabolomics approach was performed on susceptible and resistant cultivars of soybeans infected with *Sclerotinia sclerotiorum*. The results of this study showed that antifungal activity increased in the resistant cultivar by reprogramming the phenylpropanoid pathway^18^. Similarly, an untreated liquid chromatography-mass spectrometry (LC–MS) metabolomics strategy was performed to investigate metabolome alterations in anthracnose-causing *Colletotrichum sublineolum*^19,20^. Additionally, gas chromatography-electrospray tandem mass spectrometry (GC-ESI–MS/MS) was used in wheat crops infected with *Septoria nodorum,* and the concentration of secondary metabolites was found to be 200 higher in the mutant strain than in the wild-type^21^. Metabolomics is useful in plant studies since it offers the ability to identify biochemical changes relatively quickly, usually before any overt phenotypic changes become apparent^10^.

Plant-pathogen relationships are extremely interesting in terms of both biological importance and metabolite richness, and thus they are an ideal area for exploration using metabolomics techniques^10^. LC–MS is a sensitive tool for metabolic profiling that perfectly compensates for this defect and has become an important research method in the field of metabolomics^22,23^. LC–MS-based analyses aim to compare multiple biological groups to detect metabolites that have changed significantly after pathogen infection. In the present work, LC–MS was used to study the response of metabolites in *T. controversa*-infected grains (galls) and normal grains. In this study, clear differences were observed in the metabolic profiles of wheat grains after *T. controversa* infection.

## Materials and methods

### Plant material and pathogen inoculation

Wheat (*Triticum aestivum* L.) cv Dongxuan 3 seeds were collected from the Institute of Plant Protection, Chinese Academy of Agricultural Sciences, China, and all methods were performed in accordance with their relevant regulations. Wheat seeds were sterilized with 30% sodium hypochlorite for 5 min, rinsed 5 times with ddH_2_O, and germinated for 30 days in an incubator (AUCMA, Qingdao, China) to vernalize. After vernalization, seedlings were grown in a 1:2 mixture of organic matter (peat moss, Beijing, China) and soil (Beijing, China) in pots. The pots were kept in an incubator (AUCMA, Qing Dao, China) under a 14 h light:10 h dark (8–10 °C, 70% humidity) regime, and the temperature increased to 20 °C during the boot stage. The pathogenic fungus *T. controversa* was a gift from Blair Goates (the United States Department of Agriculture (USDA), Agricultural Research Service (ARS), Aberdeen, Idaho, USA). *T. controversa* was propagated in soil agar medium (20 g of agar powder, 75 g of soil in one liter of distilled water) and autoclaved to pour the media into sterilized plates. After pouring, the plates were incubated at 5 °C in a 24 h light incubator (MLR 352H, Panasonic, USA) after covering with parafilm for 60 days of incubation. Mycelium production was observed under an automated inverted fluorescence microscope (IX83, Olympus, Japan). Hyphae were collected with distilled water and used to inoculate wheat plants. During the boot stage, hyphae were injected into the spike with a syringe. The hyphae were inoculated at 8:00 am and 8:00 pm (5 ml per spike) and consecutively for 5 days, while only ddH_2_O was used for control plants. The pathogen-infected grains (galls) and normal grains were harvested when they both mature, quickly dipped in liquid nitrogen and stored at − 80 °C for further use. Nine replications of each treatment were used for reproducibility.

### Sample processing

Metabolites were extracted from 50 mg of crushed grains using ultrapure water (Watsons, China). The choice of extraction method is an important factor in any metabolomics study. The grains were crushed by following the method developed in our laboratory. Briefly, two to three grains were dipped into 2 ml Eppendorf tubes containing an appropriate amount of sterilized steel balls (1.5 mm) ground in a grinder machine (FastPrep 24 5G, MP Biomedicals, USA) for 1 min at 70 Hz for crushing. Every sample was ground 3 times for better results. Powdered tissue samples (50 mg) were first mixed with 500 µl of ultrapure water (50% methanol) and sonicated for 10 min. After this time, 50 µl of a homogenate mixture was added to 450 µl of precipitant containing internal standard (50 ng/ml propranolol for positive ion mode and 50 ng/ml tolbutamide for negative ion mode). The samples were vortexed for 1 min and centrifuged at 13,000 rpm for 10 min, and then transferred to a new sterilized Eppendorf tube and concentrated in a speed vacuum (Songyuan, Beijing, China) at 30 ℃ and the dried extract was re-dissolved of 100 µl in 50% methanol (v/v) for LC–MS analysis.

### LC–MS analysis

LC–MS analysis of the precipitant was performed in 450 µl of a methanol–acetonitrile mixture, using Dionex Ultimate 3000 (Thermo Fisher, the USA) with a Thermo Syncronis C18 column (ACQUITY BEH C18 1.7 µm, 2.1 × 50 mm) and Thermo Q EXACTIVE (Thermo Fisher, USA). After a 5 µl sample injection, chromatographic analysis was achieved with a liquid phase of 2 mmol/l ammonium acetate and 0.1% formic acid in water (A) and acetonitrile (D). The detailed gradient elution program was as follows: 95% (A) and 5% (D) to 0–2 min, 5% (A) and 95% (D) to 2–42 min, 5% (A) and 95% (D) to 42–47 min, 95% (A) and 5% (D) to 47–47.10 min, and 95% (A) and 5% (D) to 47.10–50 min.

The parameters were as follows. Ion source: ESI( ±); Monitoring mode: full scan and full MS/dd-MS^2^; Spray voltage: 3000 V; Evaporation temperature: 350 ºC; Sheath gas: 35Arb; Auxiliary gas: 10Arb; Capillary temperature: 320 ºC; S-lens RF: 80. Full scan, resolution: 70,000; AGC target: 1e^6^; Maximum TT: 100 ms; scan range: 100–1500 m/z. Full MS/dd-MS^2^, resolution: 35,000; AGC target: 1e5; Maximum TT: 50 ms; NCE: 20, 40, 60.

### Data processing

All LC–MS data were further filtered by using the R platform loaded with the xcms tool kit, including peak matching, retention time correction, variable integration (integrating the overall contribution of each variable) and data standardization (nontarget metabolite data processing). The raw data were preprocessed by noise reduction, baseline correction, peak alignment, standardization, and scaling and then analyzed by multivariate analysis, including principal component analysis (PCA), orthogonal partial least squares discriminant analysis (OPLS-DA) and hierarchical cluster analysis (HCA) with MetaboAnalyst 5.0 software (https://www.metaboanalyst.ca/faces/ModuleView.

xhtml). Metabolic pathways were further analyzed using KEGG (http://www.genome.jp/kegg/)^24^.

## Results

### Metabolite separated by LC–MS

**Figure 1:**
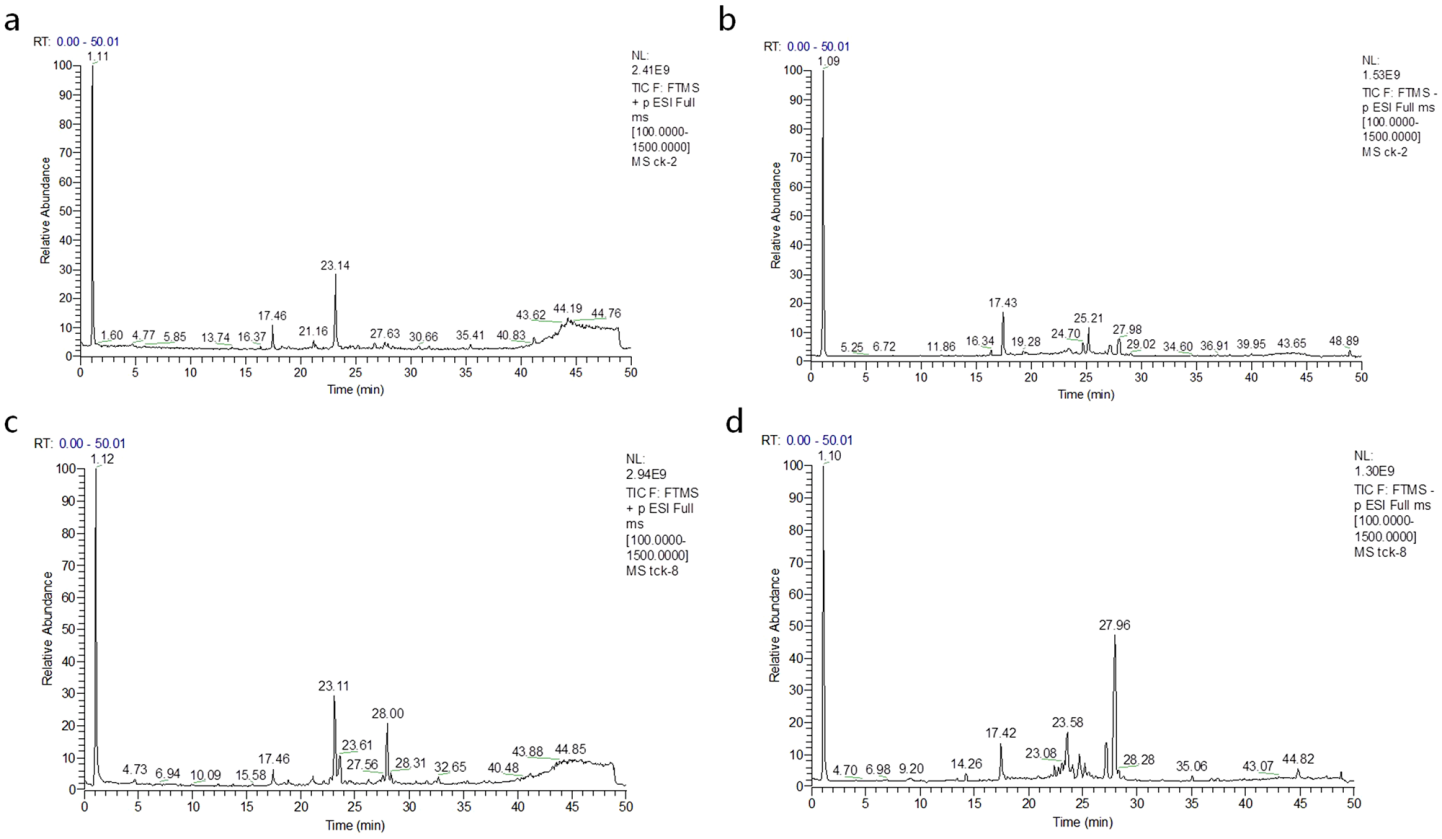
LC–MS chromatograms of the wheat grain metabolites (X-axis = time and Y-axis = response. (**a**) Relative abundance of positive ions at different time intervals for control. (**b**) Relative abundance of negative ions at different time intervals for control. (**c**) Relative abundance of positive ions at different time intervals for infected samples. (**d**) Relative abundance of negative ions at different time intervals for infected samples.

The spike traits of normal and infected by *T. controversa* were showed in Fig. S1. The metabolites separated by chromatography continuously entered the mass spectrometer for data collection. The total ion strength was obtained by adding all the ionic strengths in each mass spectrum. The peak area of endogenous substances was obtained for each sample. The chromatograms of the positive and negative ions of wheat grains are shown in Fig. 1. The total ion chromatograms of positive ions (Fig. 1a,c) and negative ions (Fig. 1b,d) of control and infected wheat were obtained by using high-resolution mass spectrometry. The significant differences based on the VIP values and the presented upregulated and downregulated metabolites were shown in Table 1, there were 62 metabolites were got totally.

**Table 1:** List of metabolites from *T. controversa* infected and non-infected wheat grains (VIP > 1).

Number	Metabolites	VIP value	Fold change	p-value
1	(9S,10E,12Z,15Z)-9-Hydroxy-10,12,15-octadecatrienoic acid	12.679	6.69 ↑	0.0000
2	l-Valine	10.858	0.63 ↓	0.0000
3	l-Glutamine	7.2423	3.03 ↑	0.0000
4	9,12,13-TriHOME	6.4916	0.14 ↓	0.0078
5	Dodecanoylcarnitine	6.438	53.30 ↑	0.0000
6	Malic acid	6.2193	0.42 ↓	0.0000
7	Clupanodonic acid	5.1969	8.13 ↑	0.0000
8	Pidolic acid	4.9952	3.00 ↑	0.0000
9	9-HODE	4.3995	1.74 ↑	0.0000
10	Androsterone	3.1927	201.55 ↑	0.0000
11	Succinylcholine	3.1339	19.62 ↑	0.0000
12	Glycerophosphocholine	3.103	3.99 ↑	0.0000
13	Nervonic acid	2.9982	67.49 ↑	0.0002
14	l-Arginine	2.7727	5.92 ↑	0.0000
15	3,8″-Binaringenin-7″-O-beta-glucoside	2.7681	0.65 ↓	0.0015
16	d-Sorbitol	2.7338	46.46 ↑	0.0000
17	Maokonine	2.7103	0.005 ↓	0.9372
18	Caffeic acid	2.6764	457.39 ↑	0.0000
19	l-Proline	2.6358	0.34 ↓	0.0000
20	Allysine	2.3258	0.73 ↓	0.0053
21	l-Leucine	2.2687	1.40 ↑	0.0000
22	cis-4-Hydroxymethylproline	2.2432	0.66 ↓	0.0230
23	Alloisoleucine	2.2421	1.39 ↑	0.0000
24	Pyroglutamic acid	2.2176	14.03 ↑	0.0000
25	d-Glucose	2.2036	0.27 ↓	0.0004
26	Nicotinamide	2.1041	0.79 ↓	0.0012
27	Sphinganine	1.9169	0.04 ↑	0.5134
28	l-Asparagine	1.8431	2.96 ↑	0.0001
29	Phenyl pyruvic acid	1.7164	22.66 ↑	0.0000
30	d-Arabitol	1.6904	19.94 ↑	0.0000
31	Adenine	1.6198	0.31 ↑	0.0010
32	Cuscohygrine	1.6038	0.04 ↓	0.2115
33	Fumaric acid	1.5928	0.47 ↓	0.0000
34	l-Phenylalanine	1.5695	1.95 ↑	0.0000
35	l-Histidine	1.552	2.60 ↑	0.0000
36	Phenylalanine	1.5408	1.90 ↑	0.0000
37	p-Aminobenzoic acid	1.5355	2.27 ↑	0.0000
38	Aminoisobutyric acid	1.4955	0.28 ↓	0.0010
39	3-Aminobutanoic acid	1.4917	0.28 ↓	0.0007
40	Cellobiose	1.4844	0.96 ↓	0.0000
41	Cucurbic acid	1.4785	0.32 ↓	0.0000
42	l-Carnitine	1.3931	9.86 ↑	0.0000
43	Coronaric acid	1.3284	2.45 ↑	0.0000
44	Tetracosanoic acid	1.327	33.30 ↑	0.0001
45	Maltol	1.3163	0.82 ↓	0.0000
46	gamma-Guanidinobutyric acid	1.2612	0.53 ↓	0.0000
47	10-Gingediol	1.2581	0.67 ↓	0.0000
48	4-Guanidinobutanoic acid	1.2481	0.53 ↓	0.0000
49	3-Hydroxyproline	1.2312	0.09 ↓	0.0647
50	Tetradecanoylcarnitine	1.2295	30.89 ↑	0.0000
51	gamma-l-Glutamyl-glutamine	1.2055	3.92 ↑	0.0001
52	Adenosine	1.2029	0.43 ↓	0.0001
53	Neuraminic acid	1.1986	0.46 ↓	0.0001
54	l-Tyrosine	1.1736	2.41 ↑	0.0000
55	l-Lysine	1.1325	3.23 ↑	0.0000
56	Prostaglandin D3	1.1192	3.60 ↑	0.0000
57	Azelaic acid	1.0857	0.62 ↓	0.0000
58	Palmitic acid	1.0643	0.72 ↓	0.0010
59	l-Tryptophan	1.0639	1.03 ↑	0.0000
60	Pregabalin	1.0448	0.99 ↓	0.0000
61	10-Gingerol	1.0388	0.48 ↓	0.0000
62	Stearic acid	1.0246	0.77 ↓	0.0082

↑ indicates the up-regulation and ↓ indicate the down-regulation of the metabolite.

### PCA of *T. controversa*-infected and control samples

**Figure 2:**
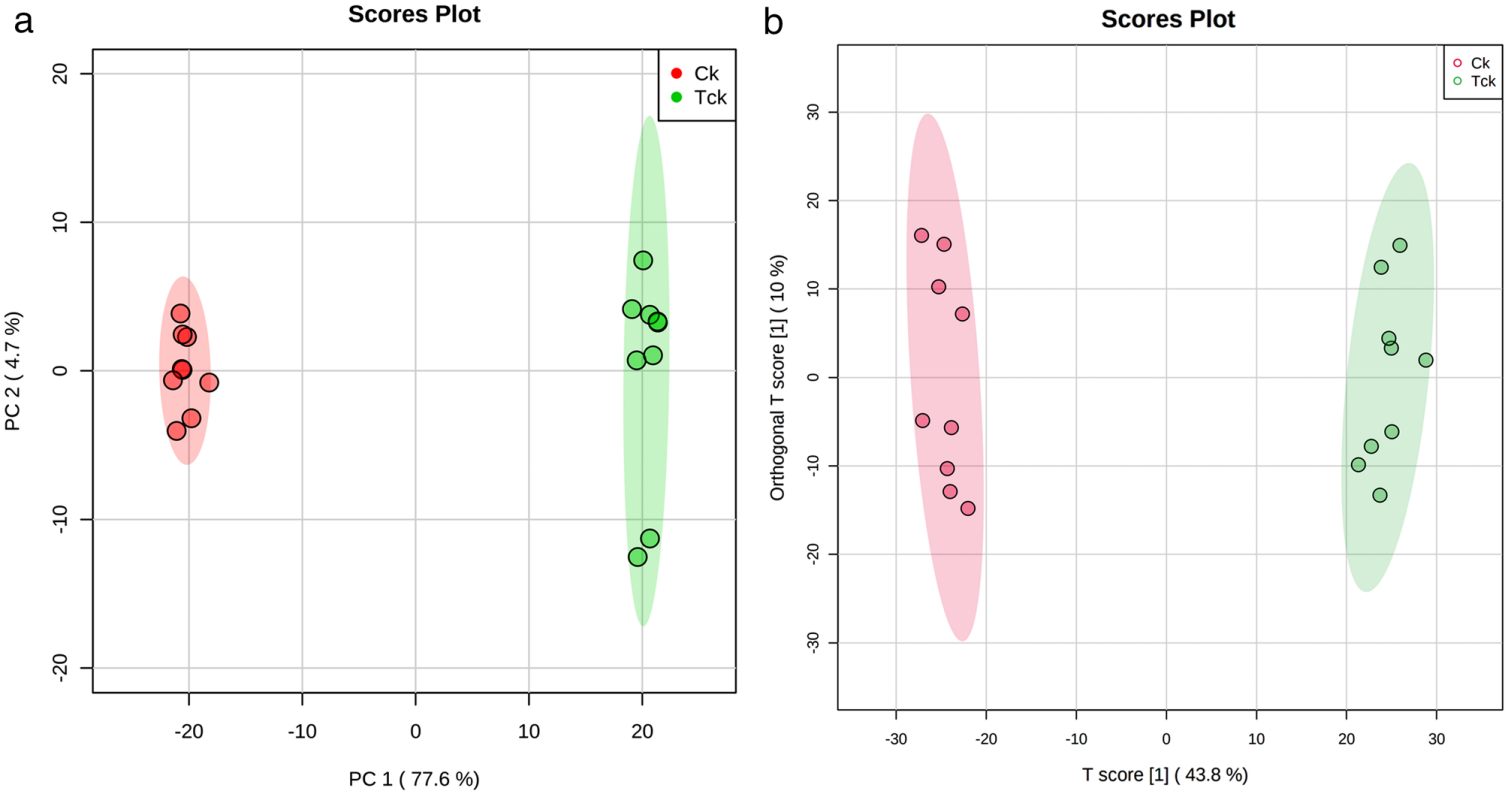
PCA and OPLS-DA analysis of *T. controversa*-infected and noninfected samples. (**a**) PCA of *T. controversa*-infected and noninfected samples. (**b**) OPLS-DA analysis (Q  = 0.938). Ck stands for noninfected samples, while Tck stands for *T. controversa*-infected samples.

PCA score plot exhibited good clustering between *T. controversa*-infected and noninfected samples (Fig. 2a,b). The results showed that the distribution of *T. controversa*-infected and noninfected metabolites was significantly different. The samples of *T. controversa*-infected were near each other, and the noninfected samples were located together, indicating that there was good reproducibility among the biological replicates of the same treatments but differences between the treatments (Fig. 2a), with R2 (0.998) and Q2 (0.997), and the first 2PCs contribution was 77.6% and 4.7%, respectively. The OPLS-DA plot (R2, 0.989; Q2, 0.968) also revealed that the samples of *T. controversa* were mainly distributed on the right side and noninfected samples were distributed on the left side, indicating that there was good reproducibility among the biological replicates of the same treatments but differences between the treatments (Figs. 2b, S2). A receiver operating characteristic (ROC) curve was generated by using Monte Carlo cross validation (MCCV) under balanced subsampling. In each MCCV, two-thirds of the samples were used to assess the importance of features. Our results showed that the differential metabolites with variable importance in the projection (VIP) values > 1 were successfully detected under the ROC curve (Fig. S3). The list of metabolites and their significant differences based on the VIP values are shown in Table 1. According to the results presented in Table 1 and in Fig. 2b, 62 differential metabolites were found. Out of these 62 metabolites, 34 were upregulated and 28 were downregulated. Out of the 34 upregulated metabolites, 12 were related to amino acids, 10 were organic acids, 3 were alcohols, 1 was a nucleoside and 8 other classes were identified. Similarly, out of 25 downregulated metabolites, 5 were related to amino acids, 10 were organic acids, 2 were fatty acids, 1 was an alcohol, 3 were sugars, 1 was a nucleoside and there were 6 other metabolites. The upregulated and downregulated metabolites are listed in Table 1. Some of the above up- and downregulated metabolites play comprehensive roles in plant pathogen interactions. For example, caffeic acid (upregulated by 457-fold) has a strong ability to scavenge peroxy free radicals after plant pathogen infection^25^. Phenylalanine metabolism (upregulated by 22-fold) will result in an increase in lignin monomers, thereby solidifying the cell wall of plants when exposed to pathogens^26^. Pyroglutamic acid (upregulated by 14-fold) suppresses certain pathogen interactions^27^. Tetracosanoic acid (upregulated by 33-fold) is also related to antifungal activity^28^.

### Screening and statistical analysis of differential metabolites

**Figure 3:**
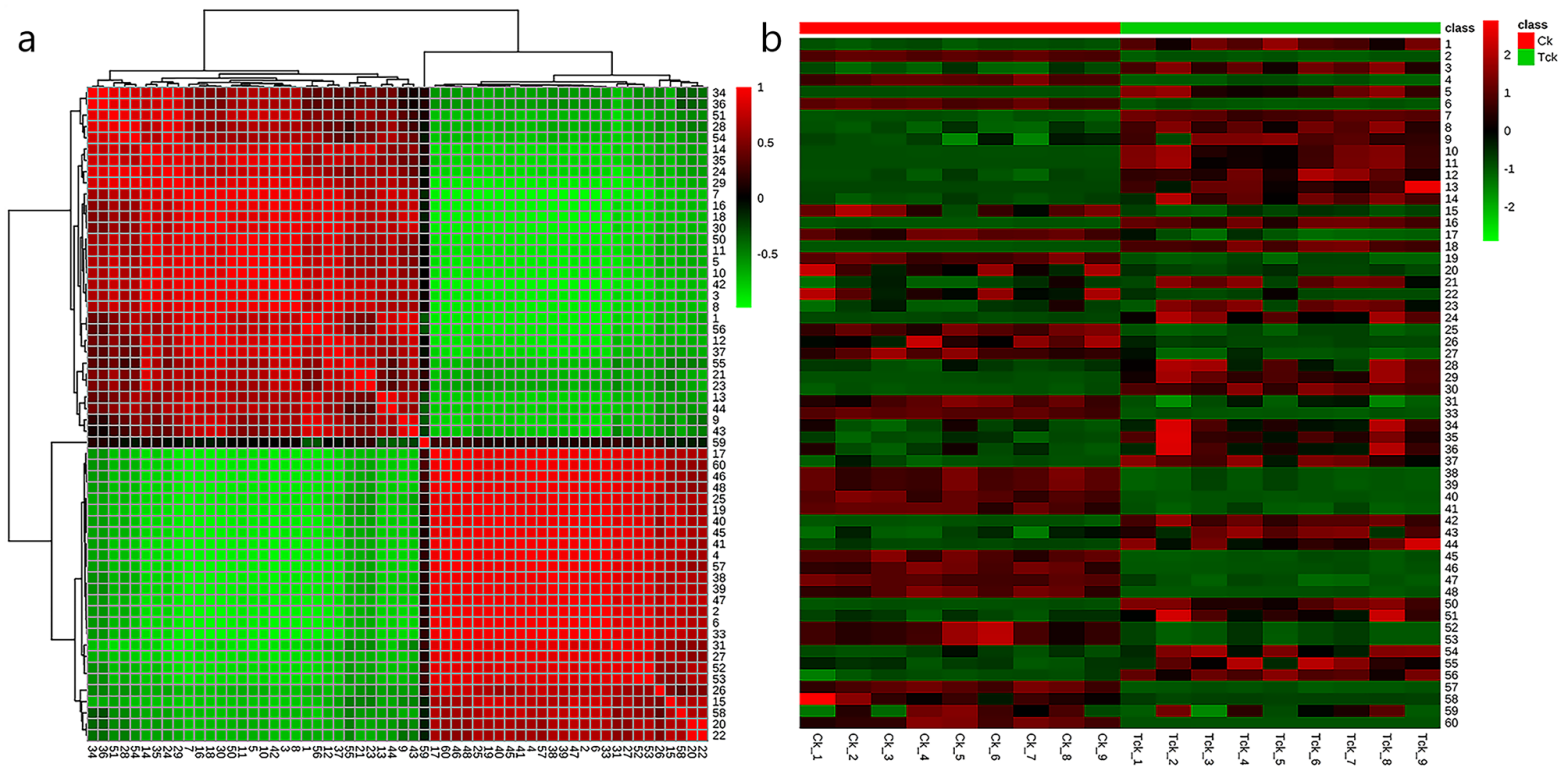
The hierarchical clustering heatmap visualizing the changes in the contents of potential metabolites in *T. controversa*-infected and noninfected samples by MetaboAnalyst 5.0 software (https://www.metaboanalyst.ca/faces/ModuleView.xhtml). (**a**) Correlation analysis of differential metabolites. Deeper colors had stronger correlations, and lighter colors had weaker correlations. (**b**) Hierarchical cluster analysis of differential metabolites. Red indicates that the contents of the metabolites increased, and blue indicates that the content of the metabolites decreased.

The correlation between the *T. controversa*-infected and non-infected samples was calculated to gain a better understanding. Red indicates a positive correlation, while green indicates a negative correlation. The results showed that metabolites in the same branch along the horizontal axis had the strongest positive correlation (Fig. 3a). The HCA results showed that 9-HODE (9) and Prostaglandin D3 (56) were positively correlated, indicating which involved in the pathogenic process of *T. controversa*. Besides, 9-HODE (9) were negatively correlated with many metabolites, such as malic acid (6), Fumaric acid (33) and Azelaic acid (57). Similarly, Prostaglandin D3 (56) were also negatively correlated with many metabolites, such as malic acid (6), Fumaric acid (33) and Azelaic acid (57). Additionally, the metabolites between infected and control samples were investigated by heatmap analysis, in which the horizontal and vertical axes represent the sample and variable information, respectively. Red indicates that the concentration of metabolites increased, green indicates that the concentration of metabolites decreased, and dark indicates that the change in metabolite concentration increased (Fig. 3b) As we saw from Fig. 3b, 9-HODE (9) and Prostaglandin D3 (56) was increased and malic acid (6), L-Proline (19) and Fumaric acid (33) were decreased in all infected groups, suggesting the invasion of pathogen will affect the normal metabolism of plants. Besides, the upregulated of antifungal substance, like caffeic acid (18), Pyroglutamic acid (24) and Tetracosanoic acid (44), were increased in infected groups, indicating they play a role in responding to the invasion of *T. controversa*.

### Metabolic pathways analysis

**Figure 4:**
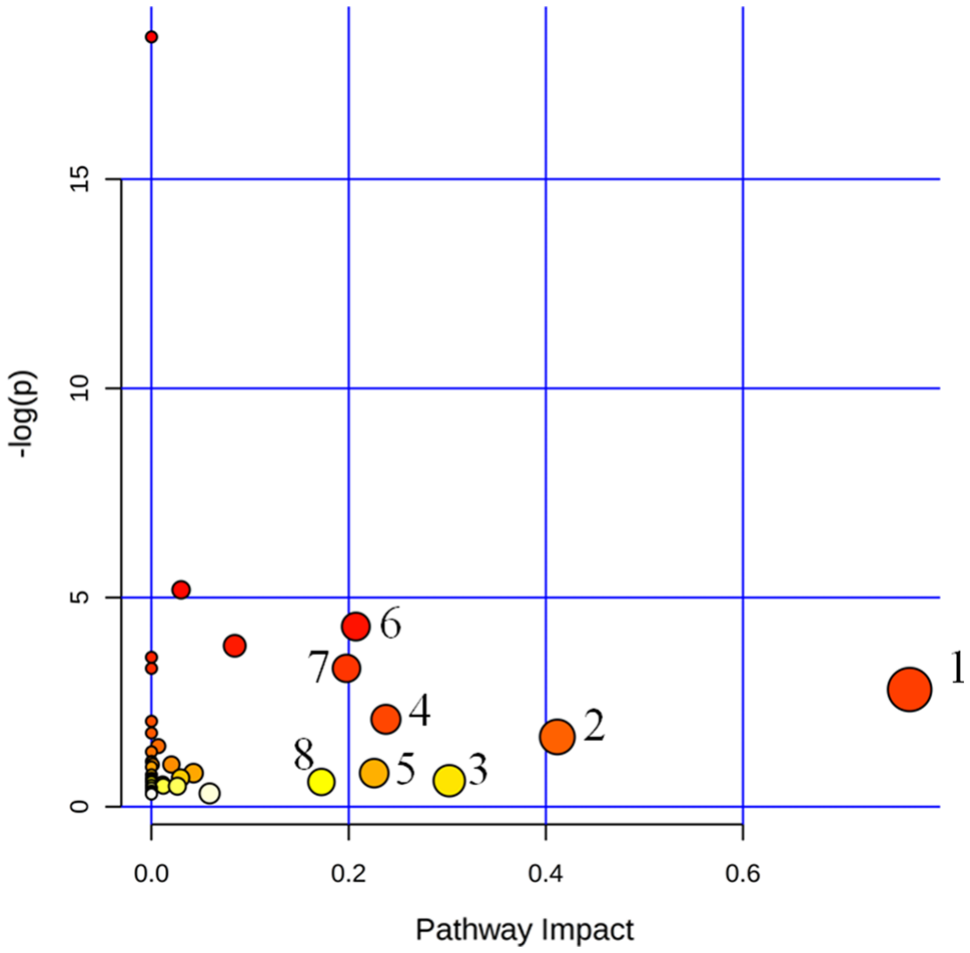
Pathway analysis overview showing the altered metabolic pathways in *T. controversa*-infected and noninfected samples based on KEGG (https://www.kegg.jp/kegg/kegg1.html) pathway analysis^24^. **1** phenylalanine metabolism; **2** isoquinoline alkaloid biosynthesis; **3** starch and sucrose metabolism; **4** tyrosine metabolisms; **5** sphingolipid metabolisms; **6** arginine and proline metabolism; **7** alanine, aspartate and glutamate metabolism; and **8** tryptophan metabolisms. A pathway with an impact value greater than 0.1 was considered a potential target.

By investigating the influence coefficient of the metabolic pathways, the pathways with impact values greater than 0.1 was found to be a potential target pathway. As a result, phenylalanine metabolism, isoquinoline alkaloid biosynthesis, starch and sucrose metabolism, tyrosine metabolism, sphingolipid metabolism, arginine and proline metabolism, alanine, aspartate and glutamate metabolism and tryptophan metabolism were closely related pathways in this study (Table 2, Fig. 4).

**Table 2:** List of metabolic pathways activated in the grains of *T. controversa* infected and non-infected.

Metabolic pathways	Total	Hits	FDR	Impact
(1) Phenylalanine metabolism	12	2	0.0608	0.7221
(2) Isoquinoline alkaloid biosynthesis	6	1	0.1888	1.0000
(3) Starch and sucrose metabolism	22	1	0.5378	1.0000
(4) Tyrosine metabolism	18	2	0.1239	1.0000
(5) Sphingolipid metabolism	17	1	0.4486	1.0000
(6) Arginine and proline metabolism	28	4	0.0135	0.4274
(7) Alanine, aspartate and glutamate metabolism	22	3	0.0367	0.4975
(8) Tryptophan metabolism	23	1	0.5539	1.0000

## Discussion

Wheat is a major staple food crop worldwide. Wheat crops have evolved efficient mechanisms to inhibit the negative effects of pathogen attack^29^. The critical difference between plants that become diseased or remain healthy after pathogen infection is the recognition of elicitor molecules released by pathogens^30,31^. The earliest response in plants after pathogen infection is due to the oxidative burst that can trigger hypersensitive cell death. This is called the hypersensitive response (HR) and is a basic cellular response following successful pathogen recognition in plants and a major element of plant disease resistance^32^. Therefore, any change in the primary and secondary metabolites of plants in response to pathogen infection may thus be a key difference in successful defense against pathogens.

Proline is a multifunctional amino acid that confers resistance against plant pathogens^33,34^ and abiotic factors^35,36^. Resistant plants increase proline accumulation against pathogen infections and vice versa in susceptible plants^33^. Similarly, cucurbic acid, a compound similar to jasmonic acid (JA), is actively involved in defense mechanisms and tuberization in different crops^37^. Our results showed that the concentrations of proline, hydroxyproline, hydroxymethylproline and cucurbic acid decreased after *T. controversa* infection (Table 1). Decreasing proline content is a common reaction, and energy demand increases for cross-signaling in plant pathogen interactions. It is also possible that elicitor molecules that are not recognized by susceptible plants cause decreases in proline and cucurbic levels, which can facilitate pathogen infection. Prostaglandin molecules act in a multidimensional way in pathogens and are used as signaling molecules to induce pathogen infection^38^. The concentration of prostaglandin increased during pathogen infection, which resembled our results (Table 1). A previous study revealed that silencing of the triple-*ppo* (this gene has a role in prostaglandin production) mutant of *Aspergillus fumigatus* was more virulent than wild-type *A. fumigatus* in an infected murine test model, and its mutant showed higher resistance than wild-type *A. fumigatus* against reactive oxygen species (ROS) produced by plants for a strong defense system after *A. fumigatus* infection^39^. Additionally, prostaglandin has a role in increasing other fungal metabolites, including tyrosine, phenylalanine and other secondary metabolites^40,41^. 9-Hydroxyoctadecaenoic acid (9-HODE) molecules are oxidation products of linoleic acid molecules that play a role in the defense mechanism, increased plant growth and promote aflatoxin B1 in *A. parasiticus* and sterigmatocystin (ST) from *A. nidulans*^42–44^. 9-HODEs also act as quorum sensing signal molecules to regulate the development and growth of *A. ochraceus*^45^. Our results showed that during *T. controversa* and wheat interactions, the concentration of 9-HODE molecules significantly increased, suggesting that *T. controversa* affects wheat grains.

In this experiment, l-phenylalanine was upregulated 1.95-fold (Table 1). In the metabolic pathways activated by *T. controversa* (Table 2), phenylalanine metabolism affects the formation and deposition of lignin, which will help plants improve immunity to fungal pathogens^26,46^. Phenylalanine ammonia lyase (PAL) is an enzyme related to wheat lignification that participates in phenylalanine metabolism by decomposing l-phenylalanine and provides precursors for lignin biosynthesis. It has been reported that the enzymatic activity of PAL in wheat resistant lines to Karnal Bunt (caused by *Tilletia indica*) was significantly higher than that of susceptible lines, which is considered to be a marker for identifying KB resistance^46^. We also found changes in starch and sucrose metabolism, which will be influenced when plants are exposed to pathogen infection, and the activation of starch and sucrose metabolism pathways can reduce the toxic substances secreted by pathogens on plants^47–50^. The activity of isoquinoline alkaloids is due to the response of plants to the invasion of pathogenic fungi, and upregulated expression of tyrosine is closely related to tyrosine metabolism, indicating that the plant signal transduction pathways are active^51,52^, as the tyrosine metabolism pathway is related to the host immune response^53^.

An important and preliminary characteristic of metabolomics data is that it can measure targeted and untargeted analysis and allows the discovery of correlations between pairs of metabolites, even when the biological connection between them is not clear. In our results, for the metabolites from the grains, a small strong correlation was noted. However, for some metabolites, there was a positive or negative correlation in the heatmap analysis (Fig. 3a,b). Additionally, the relative abundances of pathogen-infected and control groups were scattered separately, which may suggest that grain metabolites were infected by *T. controversa*. For example, metabolites that were decreased or increased after pathogen infection are involved in energy metabolism. These changes in plant metabolites may be the result of *T. controversa* infection.

## Supplementary Information

Supplementary Information.
