# Supplementary material for: Metabolomics analysis of grains of wheat infected and noninfected with Tilletia controversa Kühn
Source: Sci Rep. 2021 Sep 23;11:18876. doi: 10.1038/s41598-021-98283-3 (PMC8460654; doi:10.1038/s41598-021-98283-3)
Supplement: Supplementary file 1 — Supplementary Information. [file 41598_2021_98283_MOESM1_ESM.docx]

**Metabolomics analysis of grains of wheat infected and noninfected with *Tilletia controversa* Kühn**

Zhaoyu Ren^1+^, Mingke Fang^1,2+^_,_ Ghulam Muhae-Ud-Din^1^, Haifeng Gao^3^, Yazhen Yang^2^, Taiguo Liu^1^, Wanquan Chen^1^, Li Gao^1^*

^1^ State Key Laboratory for Biology of Plant Disease and Insect Pests, Institute of Plant Protection, Chinese Academy of Agricultural Sciences, Beijing, China

^2^ School of Agriculture, Yangtze University, Jingzhou, China

^3^Institute of Plant Protection, Xinjiang Academy of Agricultural Sciences, Ministry of Agriculture and Rural Affairs, Urumqi, China

^+^ Contributed equally to this work.

* Correspondence author: [xiaogaosx@hotmail.com](mailto:xiaogaosx@hotmail.com)


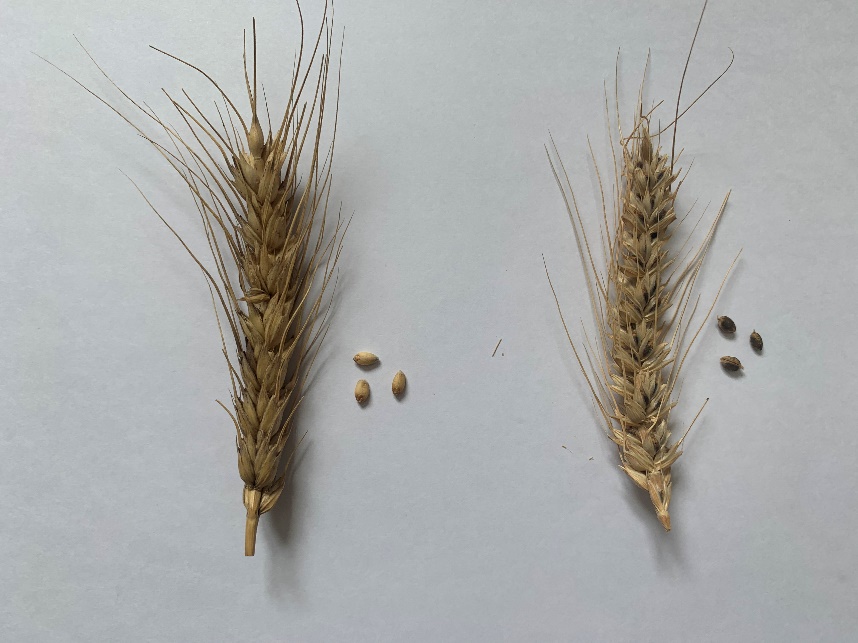


Control spike and grains Infected spike and fungus galls

**Fig. S1.** The spike traits of normal and infected by *T. controversa.* Left indicates Control spike and grains, right indicates infected spike and fungus galls.


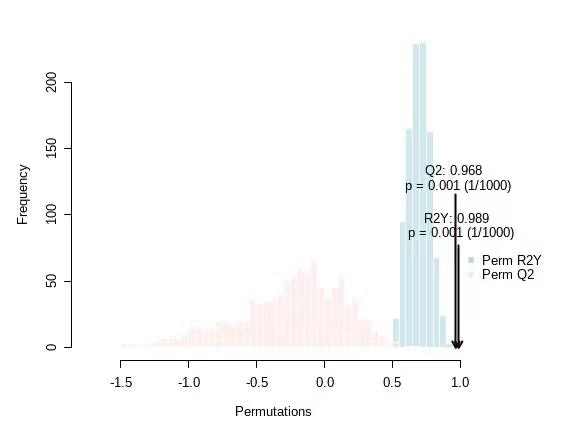


**Fig. S2.** Permutation tests of OPLS-DA. The black arrow indicates the area of OPLS-DA of this model. The P value of Q2 and R2Y is 0.001seperatively.


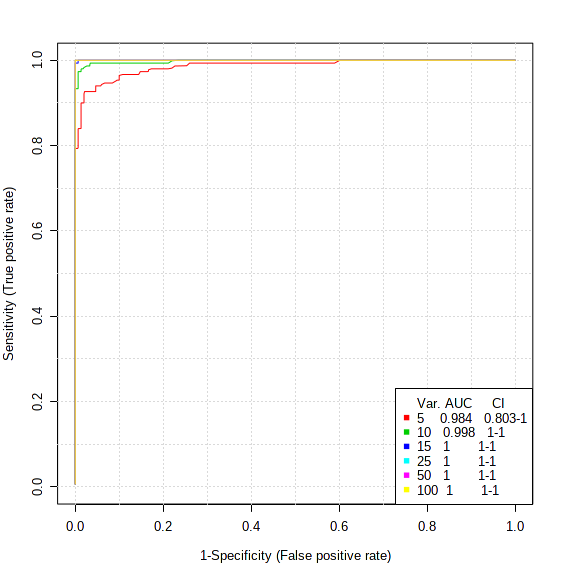


**Fig. S3.** ROC curve of metabolites in *T. controversa* infected and non-infected samples. AUC= 984 indicate that data is highly significant
